# Alpha-Mangosteen lessens high-fat/high-glucose diet and low-dose streptozotocin induced-hepatic manifestations in the insulin resistance rat model

**DOI:** 10.1080/13880209.2023.2166086

**Published:** 2023-01-18

**Authors:** Vivian Soetikno, Prisma Andini, Miskiyah Iskandar, Clark Christensen Matheos, Joshua Alward Herdiman, Iqbal Kevin Kyle, Muhammad Nur Imaduddin Suma, Melva Louisa, Ari Estuningtyas

**Affiliations:** a Department of Pharmacology and Therapeutics, Faculty of Medicine, Universitas Indonesia, Jakarta, Indonesia; b Master Program in Biomedical Sciences, Faculty of Medicine, Universitas Indonesia, Jakarta, Indonesia; c Undergraduate Program in Medicine, Faculty of Medicine, Universitas Indonesia, Jakarta, Indonesia

**Keywords:** Fatty liver, hyperglycemia, dietary fats, inflammation, metabolic syndrome, diabetes, streptozotocin

## Abstract

**Context:**

α-Mangosteen (α-MG) attenuates insulin resistance (IR). However, it is still unknown whether α-MG could alleviate hepatic manifestations in IR rats.

**Objective:**

To investigate the effect of α-MG on alleviating hepatic manifestations in IR rats through AMP-activated protein kinase (AMPK) and sterol-regulatory element-binding protein-1 (SREBP-1) pathway.

**Materials and methods:**

IR was induced by exposing male Sprague-Dawley rats (180–200 g) to high-fat/high-glucose diet and low-dose injection of streptozotocin (HF/HG/STZ), then treated with α-MG at a dose of 100 or 200 mg/kg/day for 8 weeks. At the end of the study (11 weeks), serum and liver were harvested for biochemical analysis, and the activity of AMPK, SREBP-1c, acetyl-CoA carboxylase (ACC), tumor necrosis factor (TNF)-α, interleukin (IL)-1β, IL-6, insulin receptor substrate (IRS)-1, Bax and liver histopathology were analyzed.

**Results:**

α-MG at both doses significantly lowered ALT, AST, triglyceride, and cholesterol total by 16.5, 15.7, 38, and 36%, respectively. These beneficial effects of α-MG are associated with the downregulation of the IR-induced inflammation in the liver. Furthermore, α-MG, at both doses, activated AMPK by 24–29 times and reduced SREBP-1c by 44–50% as well as ACC expression by 19–31% similar to metformin. All treatment groups showed liver histopathology improvement regarding fat deposition in the liver.

**Conclusions:**

Based on the findings demonstrated, α-MG protected against HF/HG/STZ-induced hepatic manifestations of the IR rats, at least in part *via* the modulation of the AMPK/SREBP-1c/ACC pathway and it could be a potential drug candidate to prevent IR-induced hepatic manifestations.

## Introduction

Modern lifestyles with excessive food intake, especially high-fat and high-carbohydrate foods that are not balanced with energy expenditure, will trigger the occurrence of a metabolic syndrome which is currently becoming a global epidemic (Saklayen 2018). In Indonesia, the prevalence of the metabolic syndrome is 28 and 46% in men and women, respectively (Sigit et al. 2021). It has been proven that insulin resistance (IR) contributes to the pathophysiology of metabolic syndrome (Alwahsh et al. 2017). Many studies have also demonstrated that IR is a high-risk factor for the occurrence of several chronic diseases such as type 2 diabetes mellitus, non-alcoholic fatty liver disease, and cardiovascular disease (Manco 2017; Mechanick et al. 2020). Therefore, the development of targeted therapies to treat IR is urgently needed, so that metabolic syndrome with its complications can be prevented.

Previous studies have revealed that AMP-activated protein kinase (AMPK), which is a master regulator of energy metabolism, plays a vital role in several cellular events, namely, protein synthesis, lipid metabolism, and glucose metabolism (Hardie et al. 2012; Garcia and Shaw 2017; Entezari et al. 2022). AMPK is a kinase that directly targets sterol regulating element-binding proteins (SREBPs), inhibit cleavage and nuclear translocation of SREBPs, as well as suppresses SREBPs target genes, including acetyl-CoA carboxylase (ACC) in hepatocytes exposed to high glucose levels, thereby reducing lipogenesis (Li et al. 2011).

Metformin, an AMPK activator, may decrease systemic inflammation in metabolic syndrome by decreasing C-reactive protein and interleukin (IL)-6 levels (Akbar 2003). Metformin can also alleviate liver disease and dyslipidemia due to a high-fat diet (HFD) intake by stimulating antioxidant and anti-inflammatory pathways (Yasmin et al. 2021). A previous study has also revealed that metformin can help protect pancreatic β-cells from exhaustion and decompensation and improved lipid metabolism, though it did not significantly improve IR (Sun et al. 2019; Huang et al. 2021). Until now, metformin is widely used as a drug that can increase insulin sensitivity through various mechanisms, such as decreasing hepatic glucose production and increasing glucose disposal in peripheral tissues (Wróbel et al. 2017). Besides having beneficial effects, metformin also has adverse effects that are often complained of, such as gastrointestinal disorders, vitamin B12 deficiency, and lactic acidosis (Shurrab and Arafa 2020). Therefore, drug candidates are needed that can also be used to alleviate IR-induced hepatic manifestations with the hope of well-tolerated adverse effects.

α-Mangostin (α-MG) is the main active compound of the *Garcinia mangostana* L. (Clusiaceae) fruit which is widely found in Asian countries, including Indonesia; it has been shown that α-MG exhibits anti-inflammatory, antioxidant, antitumor, anti-aging, and other biological activities (Fang et al. 2016; Lee et al. 2018; Ratwita et al. 2018). Several studies have also revealed that α-MG has various beneficial effects, including improving IR by increasing the expression of GLUT-4 and PPARγ in cardiac muscle and adipocyte (Ratwita et al. 2018, 2019), and reduced hepatic steatosis by regulating mitochondria function and apoptosis (Tsai et al. 2016). Furthermore, α-MG has been reported to increase AMPK activity and improve pulmonary fibrosis (Li et al. 2019), has neuroprotection against rotenone-induced Parkinson’s disease (Parkhe et al. 2020), suppress *de novo* lipogenesis and increase the gemcitabine response in the gallbladder carcinoma cells (Shi et al. 2020), and reduce leptin levels in olanzapine-induced metabolic disorders (Ardakanian et al. 2022). In the current study, we evaluate the effect of α-MG on hepatic manifestations of IR in rats via the AMPK/SREBP-1/ACC pathway and compare it with metformin, a well-known AMPK activator.

## Materials and methods

### Drugs and chemicals

Unless otherwise stated, all reagents were of analytical grade and purchased from Sigma-Aldrich, Indonesia. α-MG was purchased from Aktin Chemicals, Inc., Chengdu, China (batch #AM-170622, and ≥98% purity with HPLC method), whereas streptozotocin (STZ) was purchased from Santa Cruz Biotechnology, Inc. (Santa Cruz, CA, USA (catalog #sc-200719A)).

### Insulin resistance induction

IR was induced by giving a HFD (TestDiet, 58V8 rat chow, Richmond, USA) which contain by weight 46.1% fat, 35.8% carbohydrate, and 18.1% protein, with a total energy of 4.60 kcal/g, combined with 20% high-glucose (HG) drinking water for 3 weeks and a single intraperitoneal (i.p.) injection of STZ at a dose of 35 mg/kg body weight to 8- to 10-week-old male Wistar rats, which were obtained from the Laboratory Animal Center of Litbangkes, Indonesia. STZ was dissolved in 0.01 M citrate buffer (pH 4.5) and injected within 5 min of preparation. Age-matched male Wistar rats were injected with 10 µL of citrate buffer and used as non-IR-normal rats (Soetikno et al. 2020). The animals were maintained with free access to water and chow throughout the study period and were treated in accordance with the Institute of Animal Studies Ethics Committee regulations approved by our institute (ethical clearance #KET-1188/UN2.F1/ETIK/PPM.00.02/2020) and comply with the ARRIVE guideline. All efforts were made to minimize suffering.

### Experimental protocol

At 72 h after the STZ injection, the fasting blood glucose (FBG) of the rats was measured using a Glucometer 4 Accu-check, USA. The animals with FBG levels of ≥250 mg/dL were included in the study. If the FBG had not reached the target, then a second injection of STZ with a half-dose repeated once. As a result, 36 rats were divided into the following six groups (*n* = 6): (1) normal-control rats (N), (2) normal-control rats received α-MG 200 mg/kg/day (N + α-MG 200), (3) vehicle-treated HF/HG/STZ-induced IR rats (IR), (4) metformin 200 mg/kg/day-treated IR rats (IR + Met), (5) α-MG 100 mg/kg/day-treated IR rats (IR + α-MG 100), and (6) α-MG 200 mg/kg/day-treated IR rats (IR + α-MG 200). We used α-MG doses of 100 mg/kg and 200 mg/kg based on our previous study which proved that α-MG at both doses was able to increase antioxidant activity in the heart tissue of IR rats (Lazarus et al. 2020; -MG was dissolved in 1 mL of corn oil, whereas metformin was dissolved in 1 mL of 0.5% carboxymethyl cellulose and both treatments were administered orally for 8 weeks. At week 11 after the HF/HG/STZ and α-MG administration, the rats were anesthetized with a single i.p. injection of ketamine/xylazine 0.15 mL/100 g body weight and euthanized by cervical dislocation. Their liver was excised and weighed and the blood was collected, then the serum was taken and centrifuged at 1000 *g*, 4 °C, 10 min and used for biochemical parameter analyses. Half of the liver was immediately snap-frozen in liquid nitrogen and stored at −80 °C until the subsequent protein analysis. The remaining excised liver was fixed in 10% formalin and used for histopathological studies.

### Estimation of biochemical parameters

At the end of the experimental period (11 weeks), all rats have fasted for 12 h, then the blood was taken from the tail vein and collected into serum separator tubes. After the blood was allowed to sit for 30 min at room temperature was centrifuge at 1000 *g*, 10 min, 4 °C for separation of serum. Serum was stored at −80 °C until assays were performed. The serum used for the estimation of aspartate aminotransferase (AST), alanine aminotransferase (ALT), total cholesterol, and triglyceride was measured using the kits from DiaSys Diagnostic Systems GmbH (Holzheim Germany), then read using a UV/VIS spectrophotometer (PerkinElmer).

### Determination of lipid content in the liver

Hepatic lipid was extracted using a lipid extraction kit from Cell Biolabs, Inc., San Diego, CA, USA (catalog #STA-612) and were quantified using a lipid quantification kit from Cell Biolabs, Inc. (catalog #STA-613) (Seki et al. 2022).

### Gene expression analysis

Total RNA was extracted from liver tissue using a High Pure RNA Isolation kit (Roche Applied Science, Penzberg, Germany) according to the manufacturer’s instructions. RNA concentration was measured using a Nanodrop 1000 Spectrophotometer (Thermo Scientific) at a wavelength of 260 nm, followed by complementary DNA synthesis using 1 µg of RNA and the Transcriptor First Strand cDNA Synthesis kit (Roche Applied Science). The expression of genes of interest (Table 1) was analyzed using quantitative real-time PCR (qRT-PCR) using the LightCycler® 480 Instrument (Roche Applied Science) with FastStart Essential DNA Green Master Miz (Roche Life Science). All reactions were performed in the same manner: 95 °C for 10 s, followed by 45 cycles of 95 °C for 15 s and 60 °C for 1 min, and all values were normalized to the level of β-actin. The results were analyzed using the Livak method (Livak and Schmittgen 2001).

**Table 1.** List of primers used in this study.

Gene	Primers	
β-actin	Forward	5′-CTGGTCGTACCACAGGCATT-3′
	Reverse	5′-CTCTTTGATGTCACGCACGA-3′
TNF-α	Forward	5′-TCTACTCCCAGGTTCTCTTCA-3′
	Reverse	5′-CTCCTGGTATGAAATGGCAAATC-3′
IL-1β	Forward	5′-CTTGGGACTGATGCTGGTGA-3′
	Reverse	5′-TGCAAGTGCATCATCGTTGT-3′
Bax	Forward	5′-AGGGTGGCTGGGAAGGC-3′
	Reverse	5′-TGAGCGAGGCGGTGAGG-3′

### Estimation of acetyl-CoA carboxylase (ACC), sterol regulatory element-binding protein 1_C_ (SREBP-1_C_), insulin receptor substrate 1 (IRS-1), and phosphoinositide-3-kinase (PI3K) in liver tissue

Sandwich-ELISA kits for ACC, SREBP-1c, IRS-1, and PI3K (MyBioSource catalog #MBS8303295, #MBS940898, #MBS774816, and #MBS260381, USA, respectively) were performed to analyze the effect of α-MG on liver tissues.

### Western blot analysis for AMPK in liver tissue

The total protein concentration from liver tissues were measured by the bicinchoninic acid method. To determine the protein levels of AMPKα and phospho-AMPKα, equal amounts of protein extract (50 µg) were separated by sodium dodecyl sulfate-polyacrylamide gel electrophoresis (Bio-Rad, CA, USA) and transferred electrophoretically to nitrocellulose membranes. The membranes were then blocked with 5% bovine serum albumin in Tris-buffered saline Tween (20 mM Tris, (pH 7.6), 137 mM NaCl, and 0.1% Tween 20). The primary antibodies against AMPKα and phospho-AMPKα were obtained from Cell Signalling Technology, Inc. (Beverly, MA, USA) with the catalog number #CST-2532 and #CST-2535, respectively. All of the antibodies were used at a dilution of 1:1000. The membrane was incubated overnight at 4 °C with the primary antibody, and the bound antibody was visualized using the respective horseradish peroxidase-conjugated secondary antibodies (Cell Signalling Technology, Inc. (Beverly, MA, USA) catalog #CST-5127)) and chemiluminescence developing agents (Amersham Biosciences, Buckinghamshire, UK).

### Light microscopic morphological study

The samples of liver tissue were fixed with 4% paraformaldehyde for 24 h, then embedded in paraffin. Sections (5 µm thick) were stained with hematoxylin-eosin (H&E) to observe lipid accumulation in the liver.

Formalin-fixed, paraffin-embedded liver tissue sections were used for the immunohistochemical staining. After being deparaffinized and hydrated, the slides were washed in Tris-buffered saline (TBS, 10 mmol/L Tris; 0.85% NaCl, pH 7.5) containing 10% bovine serum albumin. Endogenous peroxidase activity was quenched by incubating the slides in methanol and 0.6% H_2_O_2_/methanol. For antigen retrieval, the sections were pretreated with trypsin for 15 min at 37 °C. Blocking was performed with normal rabbit serum. After overnight incubation with an anti-4-hydroxynonenal (4-HNE) antibody (Bioss, Inc., USA catalog #bs-6313-R) (1:50 dilution) at 4 °C, the slides were washed in TBS buffer, and HRP conjugated secondary antibody (Bioss, Inc. catalog #bs-0295M)) was added and incubated in the room temperature for 45 min. The immunostaining was visualized using diaminobenzidine tetrahydrochloride, and the slides were counterstained with hematoxylin. Measurement of 4-HNE was made by counting the mean number of stained cells under 400-fold magnification using a light microscope. For all sections, 25 random fields were examined per section, and 3 animals were used per group.

### Statistical analysis

Data were represented as means ± standard error of the mean (SEM). Statistical analysis of the differences between groups were performed using the One-way ANOVA followed by *post hoc* Tukey’s test using GraphPad Prism 5.0 software. Differences were considered significant at *p* < 0.05.

## Results

### Roles of α-MG on the general appearance and serum parameters in HF/HG/STZ-induced IR rats

**Figure 1.**
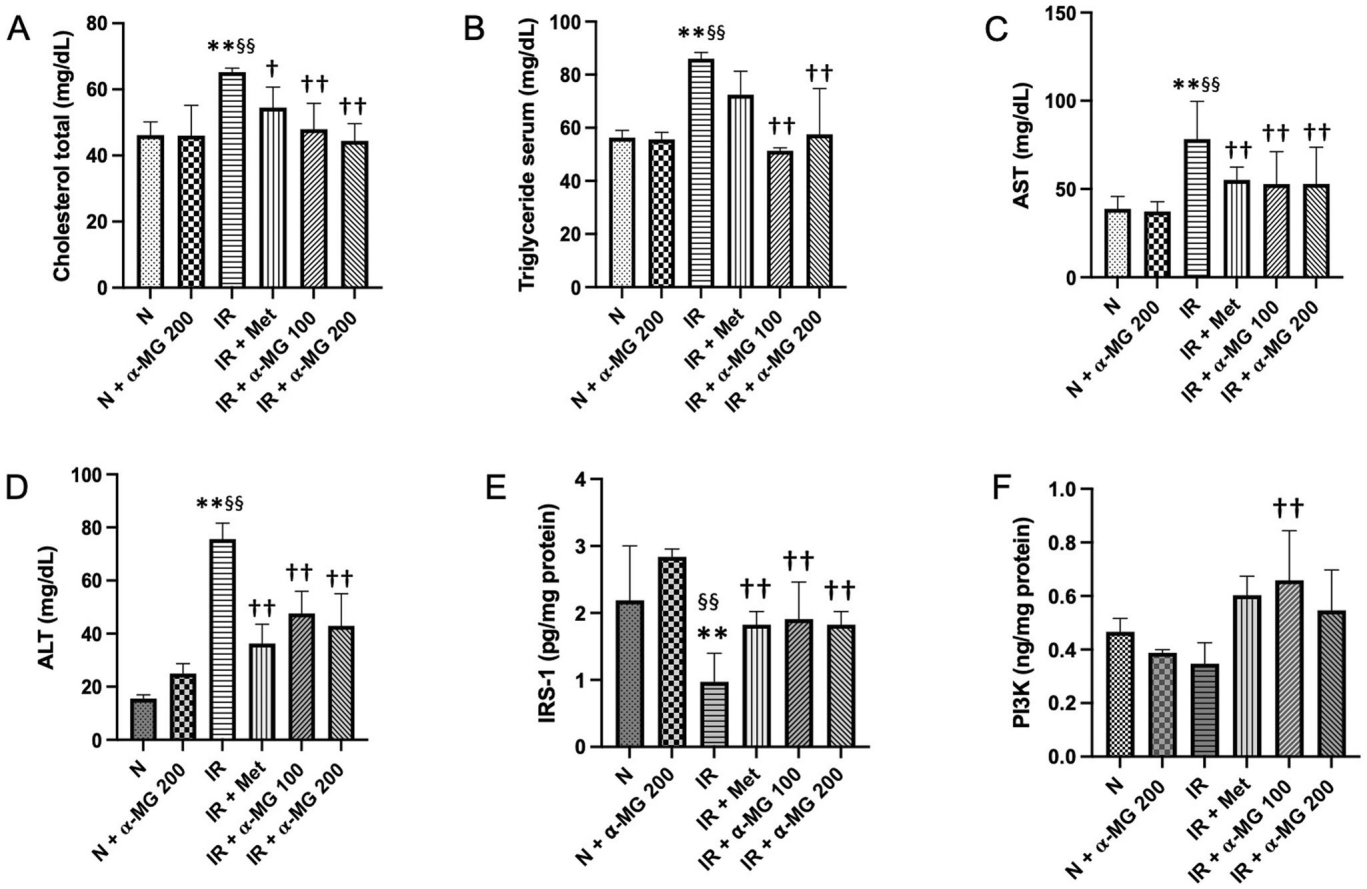
α-MG protected against high-fat/high-glucose/low-dose streptozotocin (HF/HG/STZ) induced-insulin resistance (IR) rats on hepatic manifestations such as the reduced liver lipid levels, improved liver function tests, and improved protein expression of insulin receptor substrate (IRS)-1 and phosphoinositide 3-kinase (PI3K) in the liver tissues of IR rats. The levels of liver cholesterol total (A), liver triglyceride (B), AST level in serum (C), ALT level in serum (D), protein expression of IRS-1 in the liver tissues (E), and protein expression of PI3K in the liver tissues (F). N: normal group, N + α-MG 200: normal-treated α-MG at a dose of 200 mg/kg/day group, IR: vehicle-treated insulin resistance (IR) group, IR + Met: metformin-treated insulin resistance group, IR + α-MG 100: α-MG at a dose of 100 mg/kg/day-treated insulin resistance group, IR + α-MG 200: α-MG at a dose of 200 mg/kg/day-treated insulin resistance group. Values are mean ± SD (*n* = 6). **p < 0.01 vs. N; ^§§^*p* < 0.01 vs. N + α-MG 200; ^††^*p* < 0.01 vs. IR.

At 11 weeks after HF/HG/STZ administration, the body weights (BW) of vehicle-treated IR rats were decreased considerably as compared to normal rats. In contrast, the liver weights (LW) and the LW/BW ratio of vehicle-treated IR rats, which is a surrogate marker to assess the effects of xenobiotics on specific organs, were significantly increase when compared to respective controls. Interestingly, α-MG in both doses and metformin administration could decrease LW and LW/BW ratio significantly as compared to the vehicle-treated IR rats by 19.2–24.4% and 6.9–21.6%, respectively (Table 2). In addition, administration of HF/HG/STZ leads to a pronounced increase in serum total cholesterol and triglyceride in the vehicle-treated IR rats as compared to the respective controls. On the other hand, α-MG in both doses and metformin treatment significantly suppressed the increase in both serum total cholesterol and triglyceride by 16.5–32% and 15.7–40.3%, respectively (Figure 1(A,B)). To investigate the protective effects of α-MG on HF/HG/STZ-induced IR on liver function, we measured AST and ALT in serum. The α-MG-treated rats displayed reduced AST and ALT levels compared with the vehicle-treated IR rats (Figure 1(C,D)). Next, we measured the IRS1 and PI3K in the liver tissues in all groups of experimental animals. We showed that the protein expression of IRS1 was markedly reduced in vehicle-treated IR rats compared to normal and normal-treated α-MG rats. However, there was no significant difference in the protein expression of PI3K between vehicle-treated IR rats and normal as well as normal-treated α-MG rats. The administration of α-MG at both doses could increase the protein expression of IRS1 and PI3K (Figure 1(E,F)).

**Table 2.** Biochemical parameters.

Parameters	N (*n* = 5)	N + α-MG 200 (*n* = 5)	IR (*n* = 5)	IR + Met (*n* = 5)	IR + α-MG 100 (*n* = 5)	IR + α-MG 200 (*n* = 5)
Pre-treatment body weight (pre-BW) (g)	255 ± 2	247 ± 2	254 ± 2	233 ± 2	262 ± 2	230 ± 2
Post-treatment body weight (post-BW) (g)	280.7 ± 17	273 ± 15	239 ± 15*^§^	280 ± 15^†^	276 ± 16	255 ± 15
Liver weight (LW) (g)	4.9 ± 2.2	5.2 ± 2.3	7.8 ± 2.8^**§§^	6.3 ± 2.5^††^	5.9 ± 2.4^††^	5.9 ± 2.4^††^
LW/post-BW (%)	20.5 ± 4.5	20 ± 4.5	25.9 ± 5^**§§^	24.1 ± 4.9^††^	20.3 ± 4.5	23.2 ± 4.8

Following 8 weeks of α-MG administration (100 and 200 mg/kg/day),

*p < 0.05 vs. N; **p < 0.01 vs. N; ^§^*p* < 0.05 vs. N + α-MG 200; ^§§^*p* < 0.01 vs. N + α-MG 200; ^†^*p* < 0.05 vs. IR; ^††^*p* < 0.01 vs. IR.

### Effects of α-MG on the mRNA expression of inflammatory cytokines

**Figure 2.**
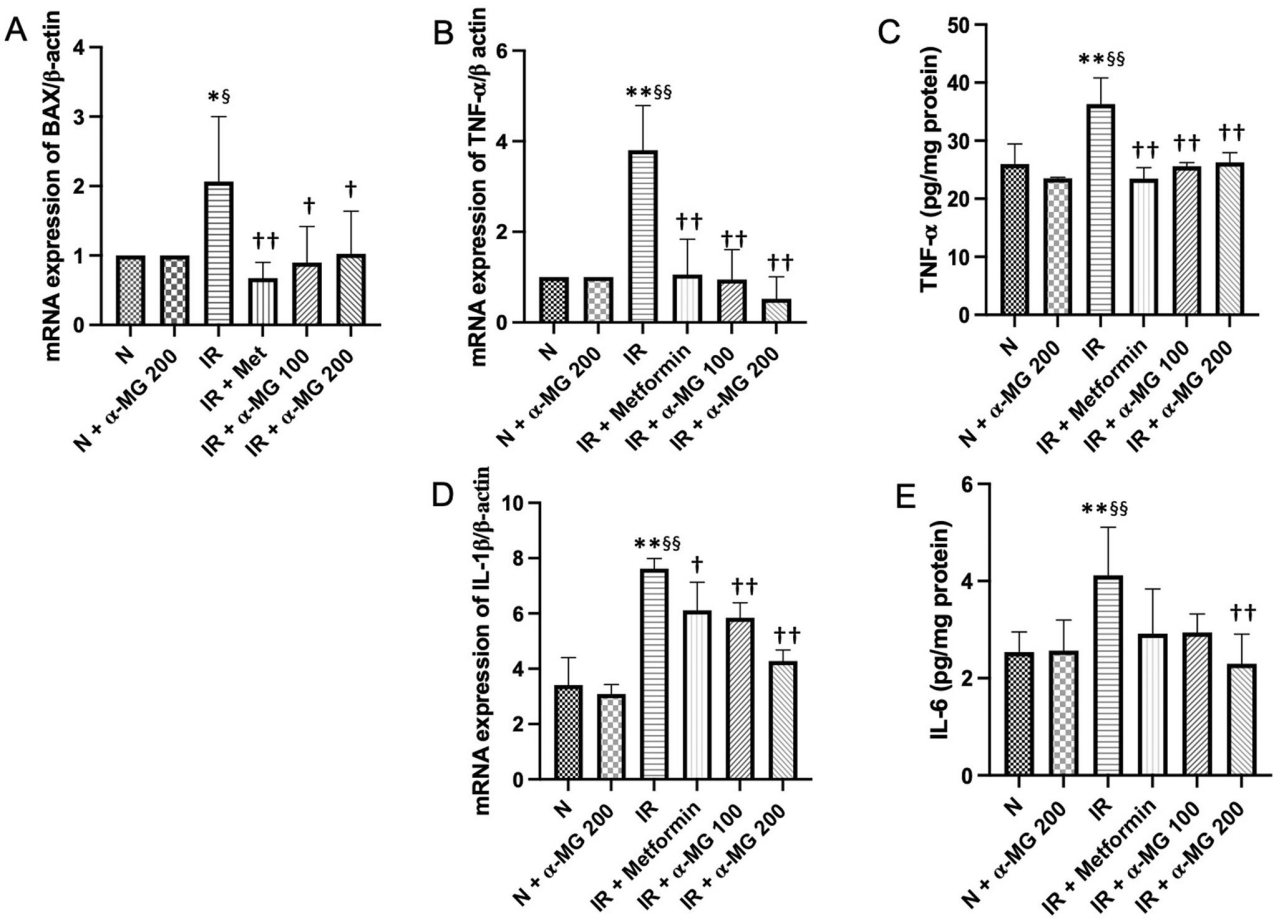
α-MG regulates high-fat/high-glucose/low-dose streptozotocin (HF/HG/STZ) induced-IR rats on inflammation process in the liver tissues. Effect of α-MG on: (A) gene expression of Bax in the liver tissues, (B) gene expression of TNF- α in the liver tissues, (C) protein expression of TNF- α in the liver tissues, (D) gene expression of IL-1β in the liver tissues, and (E) protein expression of IL-6 in the liver tissues. Values are mean ± SD (*n* = 6). *p < 0.05 vs. N; **p < 0.01 vs. N; ^§^*p* < 0.05 vs. N + α-MG 200; ^§§^*p* < 0.01 vs. N + α-MG 200; ^†^*p* < 0.05 vs. IR; ^††^*p* < 0.01 vs. IR.

Induction of inflammation has been implicated in IR-mediated hepatic disease, which was confirmed by the upregulated gene expression levels of Bax and IL-1β as well as upregulated protein expression levels of TNF-α and IL-6 in the vehicle-treated IR rats. The α-MG-treated IR rats markedly reduced the gene and protein expression of those inflammatory cytokines in their liver (Figure 2(A–E)).

### α-MG abrogates the downregulation of p-AMPK and its downstream proteins

**Figure 3.**
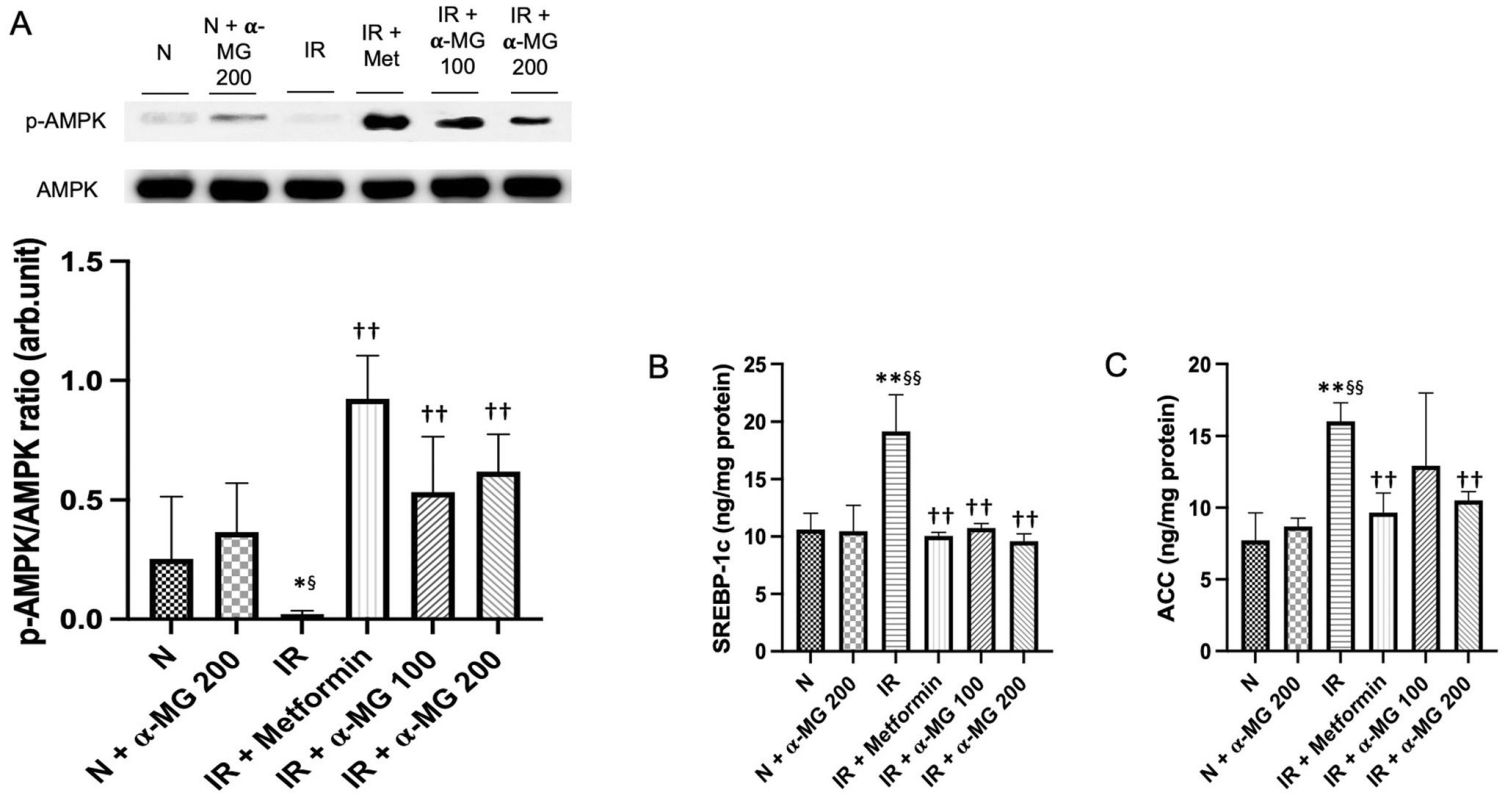
α-MG increased the expression of AMPK and decreased the expression of SREBP-1 and ACC. (A) Western blots p-AMPK and its relative contents, (B) protein expression of SREBP-1c in the liver tissues, and (C) protein expression of ACC in the liver tissues. Values are mean ± SD (*n* = 6). *p < 0.05 vs. N; **p < 0.01 vs. N; ^§^*p* < 0.05 vs. N + α-MG 200; ^§§^*p* < 0.01 vs. N + α-MG 200; ^††^*p* < 0.01 vs. IR.

It has been reported that the downregulation AMPK causes an increase in the activity of SREBP-1c and ACC which in turn leads to enhanced production and impaired catabolism of lipid and glucose in the liver which contributes to IR and dyslipidemia. In this study, we also observed a downregulated AMPK phosphorylation in the liver of vehicle-treated IR rats (Figure 3(A)). Furthermore, the vehicle-treated IR rats displayed increased SREBP-1c and ACC protein expressions. The α-MG-treated IR rats at both doses demonstrated significantly attenuated phosphorylation of AMPK and decreased SREBP-1c as well as ACC protein expressions in their liver (Figure 3(A–C)).

### α-MG reduces lipid accumulation and lipid quantification in the liver of HF/HG/STZ-induced IR rats

**Figure 4.**
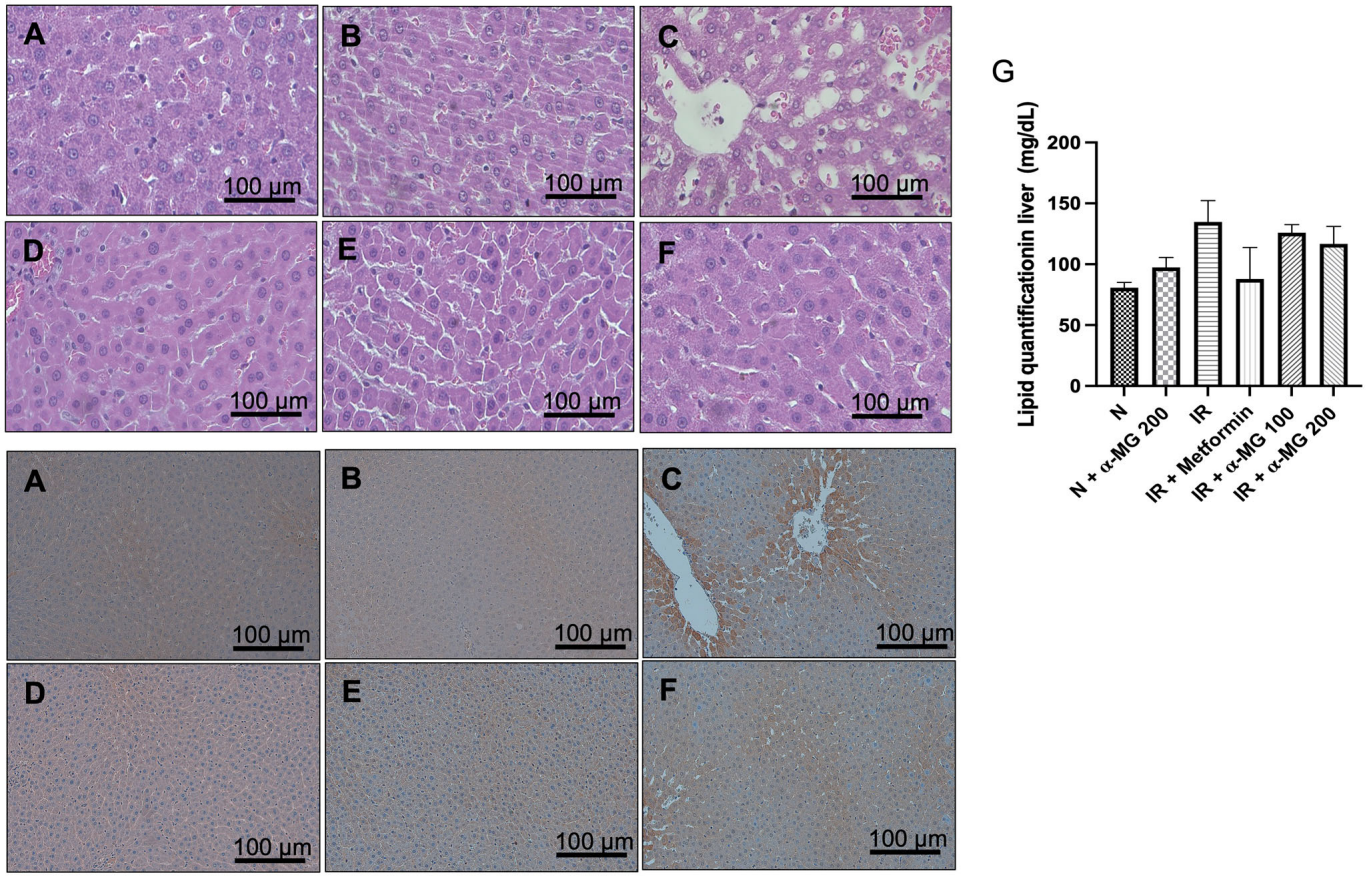
(A–F) Hematoxylin & Eosin staining of liver tissue. Lipid accumulation was marked in the IR group compared to other groups. Treatment with α-MG at 100 and 200 mg/kg/day markedly suppressed lipid accumulation (×400 magnification), (G) lipid quantification analysis in the liver tissues, (H–M) immunohistochemical staining with 4-HNE show lipid peroxidation with stained positive for 4-HNE (arrows). Treatment with α-MG at 100 and 200 mg/kg/day reduced 4-HNE (×200 magnification).

HE staining of paraffinized sections showed that the lipid accumulation in the vehicle-treated IR liver rats were more pronounced than the normal liver tissues, while lipid accumulation was markedly reduced in the groups administered with α-MG (Figure 4(A–F)). In line with HE staining, lipid quantification also showed that α-MG administration (100 and 200 mg/kg) significantly reduced lipid accumulation in the liver tissues (Figure 4(G)).

### α-MG reduces 4-HNE expression in the liver of HF/HG/STZ-induced IR rats

We performed IHC to measure the protein expression of 4-HNE, one of the stable and reliable markers of lipid peroxidation in the liver, in the normal, vehicle, and α-MG-treated IR rats. The vehicle-treated IR liver rats were found to have increased expression of 4-HNE (Figure 4(J)) in comparison to normal and α-MG-treated normal rats (Figure 4(H,I)). The metformin and α-MG administration at both doses attenuated the increased expression of 4-HNE in the liver of IR rats (Figure 4(K–M)).

## Discussion

In the Western diet, long-term consumption of high-fat and high-carbohydrate foods can lead to various diseases including obesity, IR, hypertriglyceridemia, NAFLD, and NASH, all of which are associated with inflammatory and increased oxidative stress conditions (Kim et al. 2021). Our previous study has indicated that the administration of a high-fat and high-carbohydrate diet which contains by weight 46.1% fat, 35.8% carbohydrate, and 18.1% protein, can cause IR in rats, which is characterized by an increase in Homeostatic Model Assessment for Insulin Resistance (HOMA-IR) and FBG levels (Soetikno et al. 2020). In the present study, we found that IR also causes lipid accumulation and lipid peroxidation in the liver. Treatment with α-MG at both doses significantly attenuated those alterations in the liver of rats as evidenced by decreased lipid droplets, decreased protein expression of 4-HNE, as well as attenuated inflammation in the liver, at least by modulating the AMPK/SREBP-1c/ACC cascade.

AMPK is the most important regulator of energy metabolism homeostasis both at the cellular and whole organism level, besides that AMPK also plays a crucial role in various physiological processes, including modulating inflammation and increasing insulin sensitivity in peripheral tissues (Herzig and Shaw 2018). The pharmacological function of α-MG is to activate AMPK has been revealed in various physiological processes. α-MG decreased oxidative stress in the lungs partly by activating the AMPK mediated signalling pathway (Li et al. 2019). In addition, it has been known that α-MG was able to restore leptin levels and oxidative stress in olanzapine-induced metabolic disorders in rats by increasing AMPK phosphorylation in liver (Ardakanian et al. 2022). A previous study has shown that under conditions of oxidative stress, lipid peroxidation of polyunsaturated fatty acids lead to the production of 4-HNE, which can further impair insulin action in muscle cells (Pillon et al. 2012). In accordance with previous studies, we demonstrated that α-MG at both doses increases AMPK activity and reduces oxidative stress, comparable to metformin, as shown by decreased protein expression of 4-HNE in the liver tissues of HF/HG/STZ-induced IR rats.

One of the important features of IR is the contribution of inflammation. It has been reported that increased activity of AMPK can prevent and/or ameliorate type 2 diabetes mellitus and IR by inhibiting inflammatory response (Behrouz et al. 2020; Yap et al. 2020). AMPK impedes the inflammatory process by directly inhibiting the NF-κB signalling pathway and inhibiting IKK phosphorylation (Chen et al. 2018). It has also been observed that metformin, an AMPK agonist, can diminish systemic inflammation by lessening the activity of PPAR_γ_ and normalized the SREBP-1c and fatty acid synthase mRNA in the liver of HFD fed rats (Yasmin et al. 2021). In this study, we found that α-MG as well as metformin can decrease the inflammatory response through AMPK activation and further decrease proinflammatory cytokines such as TNF-α, IL-1β, IL-6, and Bax.

It has been shown that AMPK downregulation leads to an augmentation of SREBP1c and ACC genes expression which will further increase cholesterol and fatty acid biosynthesis (Kobayashi et al. 2018). Our data demonstrated that HF/HG/STZ-mediated downregulated of AMPK and upregulated SREBP-1c and ACC protein expressions were significantly suppressed after α-MG treatment. Thus, in our study, the administration of α-MG can reduce cholesterol and triglyceride levels. Shi et al. (2020), found that in gallbladder carcinoma cells, α-MG was effective in inhibiting lipogenesis via targeting the AMPK/SREBP1. A previous study has also indicated that in HFD-fed rats, the increase in plasma cholesterol and triglyceride lead to lipid accumulation in the liver (Shin et al. 2020). In line with the previous study (Shin et al. 2020), we also demonstrated that lipid accumulation in the liver occurred in HFD-fed rats and administration of α-MG was able to reduce those conditions. In addition, we also showed that in HFD-fed rats there was an increase in AST and ALT levels; and interestingly, α-MG administration as well as metformin were able to reduce these levels to normal values. This result is in accordance with the previous study which proved that AST and ALT are associated with hyperinsulinemia and IR, independent of obesity (Esteghamati et al. 2011).

Next, we evaluated the insulin signalling pathway and its relationship to AMPK. A previous study has shown that insulin signalling and AMPK show vital roles in balancing intracellular energy levels and glucose uptake, in which both pathways stimulate energy conservation and survival of muscle exposed to severe glucose deprivation (Chopra et al. 2012). It has been demonstrated that insulin signaling is mediated by IRS protein. IRS1 is the most common and widely expressed in many tissues including the liver. Metabolic effects of insulin downstream of IRS proteins are mediated by the PI3K (Gallagher et al. 2012). In the present study, HF/HG/STZ administration was able to downregulate the level of IRS1 and PI3K as compared to that of normal rats, while the administration of α-MG at both doses and metformin showed an increase in IRS1 and PI3K. This result is consistent with evidence that α-MG activates insulin signalling and protects pancreatic β-cells against STZ-induced apoptotic damage (Lee et al. 2018).

This study demonstrates that α-MG improves the manifestations of hepatic dysfunction due to IR in rats, by modulating the AMPK/SREBP-1c/ACC signalling pathways. In this study, we also noticed that oral administration of α-MG doses of 100 mg/kg and 200 mg/kg did not show significantly different effectiveness, this may be due to the low oral bioavailability of α-MG because it undergoes extensive first-pass metabolism in the liver (Li et al. 2011). Though in this study α-MG showed promising results in preventing IR-induced hepatic dysfunction, there are several limitations. First, in the present study, the time to induce IR to lipid accumulation in the liver with a HFD was only 11 weeks, which is most likely if the time for induction is extended, lipid accumulation in the liver will be more extensive. Secondly, the use of experimental animals to induce IR by giving a HFD and STZ injection does not always resemble the conditions that actually occur in humans.

## Conclusions

This current study aimed to evaluate the effectiveness of α-MG as compared to metformin against impaired liver function in IR rats. The results of this study showed that α-MG at a dose of 100 mg/kg and 200 mg/kg is comparable to metformin and is able to prevent impaired liver function by at least increased AMPK activity and decreased expression of SREBP-1c and ACC, and as a result, attenuated IR-mediated inflammation, and lipid peroxidation, and lipid accumulation in liver tissues. Given the promising results of this study, α-MG may be a candidate for therapy to treat liver disorders caused by IR, and may be used as a substitute for metformin, although further subclinical and clinical studies are needed to prove this.
